# Evaluation of predation pressure by wild canids on threatened wild ungulates in the Hirpora Wildlife Sanctuary, Pir Panjal Range of the Lesser Himalaya

**DOI:** 10.5455/javar.2025.l946

**Published:** 2025-08-18

**Authors:** Zakir Hussain Najar, Iyaz Quyoom, Riyaz Ahmad, Abdulaziz R. Alqahtani, Bilal A. Bhat

**Affiliations:** 1 Department of Zoology, University of Kashmir, Srinagar, India; 2 National Centre for Wildlife, Riyadh, Saudi Arabia; 3 Department of Biology, College of Science, University of Bisha, P.O. Box 551, Bisha 61922, Saudi Arabia

**Keywords:** Himalayan wolf, Jacob’s index, Kashmir Himalaya, Markhor, Kashmir musk deer

## Abstract

**Objective:**

This study aims to evaluate the predation pressure of wild canids on two threatened wild ungulates, the Pir Panjal markhor (*Capra falconeri cashmeriensis*) and the Kashmir musk deer (*Moschus cupreus*) in the Hirpora Wildlife Sanctuary (HWLS).

**Materials and Methods:**

Between August 2020 and July 2022, we surveyed trails (*n =* 27) to collect scat samples of three canid species in the HWLS for dietary analysis. To determine the prey species, a sample of hair was taken from each fecal sample and compared with the available reference collection and published literature. We followed the total count method to estimate ungulate availability in the sanctuary. The selectivity of threatened wild ungulates by wild canids was assessed by Jacob’s selectivity index. The biomass contribution of prey items to canid species was determined by multiplying the dry weights of prey remnants by coefficients of digestibility.

**Results:**

The analysis revealed the presence of 10 different types of dietary items in red fox scats, while golden jackal and Himalayan wolf scats contained 11 identified items each, along with unidentified material. In all canid species, animal matter contributed more than plant matter. According to this study, livestock contributed the most to the biomass consumption of the three canid species. The Himalayan wolf also showed a small proportion of wild ungulates in its diet. According to Jacob’s selectivity index, the Himalayan wolf avoided wild ungulates, probably due to the extremely small population of these ungulates in the sanctuary.

**Conclusion:**

The local wild ungulate populations in the area have been reduced to a level where the wild canids cannot opt to prey on them because the costs would outweigh the benefits. Therefore, in order to restore the population of wild ungulates, other contributing factors need to be recognized and given due attention.

## Introduction

The Himalayas, a vast mountain range spanning several countries in South Asia, are home to a diverse variety of wildlife species. These ecosystems provide critical habitats for many species, including carnivores and ungulates, several of which are placed under threatened categories of the IUCN. The interactions between carnivores and ungulates can have significant ecological and conservation implications through the food chain and nutrient cycling [1, 2]. Carnivores play a significant role in shaping these dynamics, and their interactions with ungulates can have profound ecological impacts [3]. Predators help regulate ungulate populations, prevent overgrazing, and promote healthier ecosystems by targeting weaker or diseased individuals [4, 5]. However, excessive predation can lead to declines in ungulate populations, especially when predator numbers are unnaturally high or when human activities alter predator–prey dynamics [6]. This can directly result in a decline in the number of ungulates, especially in regions with large predator populations.

Ungulates are integral parts of these ecosystems, contributing to nutrient cycling and shaping plant communities [7, 8]. These ungulates have adapted to the challenging environment and have complex interactions with local predators and plant life. However, they also face numerous threats that can affect both their populations and the functioning of the entire ecosystem. Habitat loss and fragmentation, caused by human activities such as agriculture, urbanization, and infrastructure development, pose substantial risks to ungulates [9]. As a result of habitat loss and fragmentation, these animals encounter challenges in finding optimal habitats to secure food and shelter. Additionally, these ungulates are critical food sources for apex predators like the snow leopard (*Panthera uncia*), common leopard, and wolf [6, 10]. The existence of ungulates in viable numbers is essential for maintaining healthy predator–prey dynamics and the long-term survival of apex predators that depend on them [11].

Normally, the first step in investigating a species’ ecology is to evaluate its diet. The establishment of species and ecosystem management approaches for a particular species is greatly influenced by feeding behaviors [12]. This is because the diet directly reflects resource consumption and can shed light on habitat exploitation, as well as competitive interactions [13]. The food habits exhibited by carnivores play an important role in their ecological function, as they help determine the availability and abundance of suitable prey [14, 15]. Furthermore, these patterns are influenced by the physical, behavioral, and physiological adaptations of the predators, collectively enhancing their capacity to successfully hunt down a wide range of prey species [16].

The carnivores in the least altered Himalayan landscape primarily rely on wild ungulates as a food source [5, 17]. The wolf is recognized as a prominent predator targeting sizeable ungulates [18, 19]. Additionally, some mesocarnivores, acting as opportunistic predators, frequently choose ungulates as a food source in European countries [20]. Hence, a clear understanding of predator–prey dynamics is essential for developing effective conservation strategies. Furthermore, there is a dearth of knowledge regarding the dietary ecology of canids in the landscape, particularly about their role in the recovery of wild ungulates.

The populations of the ungulates, such as the Pir Panjal markhor (*Moschus cupreus*) and Kashmir musk deer (*Moschus cupreus*), in the sanctuary already appear to have declined below critical levels [21]. In this context, we attempted to evaluate the role of wild canids in the recovery of threatened wild ungulates in the Hirpora Wildlife Sanctuary (HWLS). We expect the Himalayan wolf to subsist on livestock when it is around, in addition to wild ungulates and other natural prey in the sanctuary. The red fox may be less dependent on wild ungulates and more reliant on smaller natural prey due to its size and feeding habits. Furthermore, we anticipate the golden jackal to be more dependent on livestock carcasses, rodents, and human subsidies because it is primarily restricted to lower altitudes close to human settlements and agricultural fields.

## Materials and Methods

**Figure 1.**
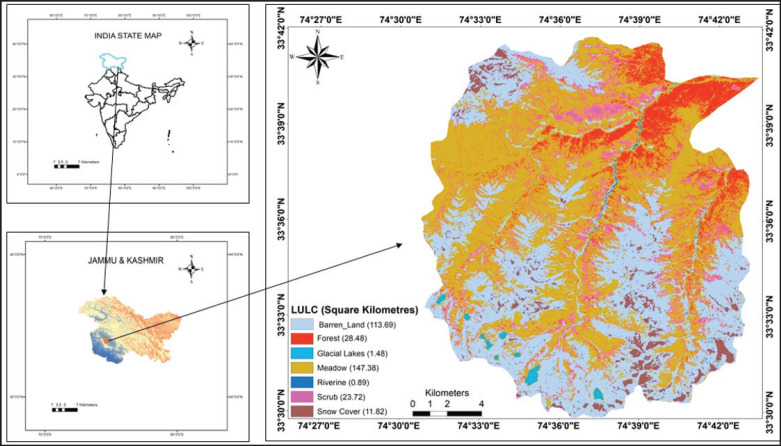
Location of Hirpora Wildlife Sanctuary, Pir Panjal range of the Lesser Himalaya.

Since the work relies on non-invasive sampling (fecal examination) and does not involve animal handling, ethical approval was not required. The HWLS is located within the Shopian district (33°29’ to 33°41’ N and 74°30’ E to 74°43’ E) of Kashmir, nestled in the Pir Panjal range of the Western Himalayas. It spans approximately 341 km^2^ (Fig. 1). The elevation varies from 2557 to 4745 m AMSL. The sanctuary is home to many important plants and animals. The Pir Panjal markhor, Kashmir musk deer, Himalayan brown bear (*Ursus arctos isabellinus*), Himalayan black bear (*Ursus thibetanus laniger*), Himalayan wolf (*Canis lupus chanco*), and red fox (*Vulpes vulpes*) are notable mammalian species [22]. The sanctuary also has a diverse bird population and is one of the top vulture sites [21, 23].

The area has different vegetation types [24]. Silver fir (*Abies pindrow*) and spruce (*Picea smithiana*) dominate exposed slopes, while blue pine (*Pinus wallichiana*) grows in drier areas at lower altitudes. Broad-leaved Himalayan maple (*Acer caesium*) grows occasionally. The most prevalent ground cover plants are Himalayan indigo (*Indigofera heterantha*), Himalayan viburnum (*Viburnum grandiflorum*), and Kashmir elder (*Sambucus wightiana*). The subalpine zone (2,100–3,200 m) is dominated by juniper (*Juniperus *sp.), Himalayan rose (*Rosa microphylla*), and rhododendron (*Rhododendron *sp.), with a considerable amount of Himalayan birch (*Betula utilis*) forming the tree line. Grass and herbs dominate alpine zones (3,200–4,600 m).

The sanctuary serves as the main route for migratory tribal communities, such as Gujjars and Bakerwals, who travel to Kashmir along with their livestock herds. This migration typically commences in mid-spring and concludes in mid-autumn. Additionally, the sanctuary serves as a grazing area for both local and migratory herders’ livestock (sheep, goats, horses, buffalo, and cows). These herder groups sustain their economies by engaging in agricultural practices and raising livestock. Key farming activities encompass the cultivation of potatoes and apple production within the designated area and its surrounding regions.

Between August 2020 and July 2022, we surveyed trails (*n =* 27) to collect scat samples of three canid species inhabiting the HWLS. Distinguishing characteristics such as scat morphology, size, diameter, content, and surrounding spoor were utilized to differentiate the scats of three canid species and those of other carnivores [25–27]. The scats were placed in paper bags labelled with relevant information, including date, location name, and habitat type. We recorded the GPS coordinates and altitude of each sample. Initially, the scats were sun-dried, followed by dehydration in an oven at a temperature of 60°C.

The collected scat samples were soaked in water for 24 to 36 h to investigate the food prey species. Subsequently, the scats were thoroughly washed using a 60 μm mesh sieve and tap water. After removing redundant water through sun drying, the fragments were then placed on a tray, and various prey items were separated and identified. To determine the prey species, a sample of hairs was taken from each fecal sample. The prey components, notably the hair, were carefully handled with alcohol before cleaning with xylene. We used the reference collection available in the Zoology Department, University of Kashmir, and previously published literature to identify the prey species [18, 28]. Hair was identified using characteristics such as medullary and cuticular scale arrangements. The remains of each prey species were weighed with an electronic balance.

Ungulate availability was estimated in collaboration with the Jammu and Kashmir Wildlife Protection Department. The estimation was held in December 2021. We followed the total count method [29, 30]. During winter and spring, the wild ungulates are confined to areas with lesser snow, which makes their counting easier. Eight teams walked on predetermined trails to scan the area with the help of binoculars. The census was aided by other research scholars from the University of Kashmir and wildlife guards from the Department of Wildlife Protection, Jammu and Kashmir. The trails were selected to maximize the visual coverage of the markhor and musk deer habitats. Each team consisted of at least two observers: one who was familiar with the area and the other who could spot and identify the species easily. Trail walks were started around sunrise when the wild ungulates become active, and binoculars were used for effective scanning. For every sighting, the species, time, group structure, location, and direction of movement were recorded. The exercise was repeated thrice with a gap of one day after every count. The teams compared their sighting data each day after the completion of the count to drop the suspected double counts.

The selectivity of threatened wild ungulates by wild canids was assessed by Jacob’s selectivity index (D) [31]:


$$D\;=\;\frac{(r-p)}{(r\;+\;p-2\mathrm{rp})}$$

where *p* represents the number of a specific ungulate species in the free-living population and *r* is the number of that prey species in canid kills. Index value ranges from −1 (complete avoidance) to +1 (preference).

Biomass contribution of prey items to canid species was determined by multiplying the dry weights of prey remnants by the coefficient of digestibility (COD) factor. The COD for ungulates is 118, for small mammals is 23, for birds is 35, for insects is 5, and for plant material is 14 [27, 32, 33].

B = *w_i_* × *q_i_*

where *w_i_* is the weight of the remnant of *i_th_* ungulate species, and *q_i_* is the COD.

Descriptive analysis was conducted to compute the maximum number of ungulates seen.

## Results

**Figure 2.**
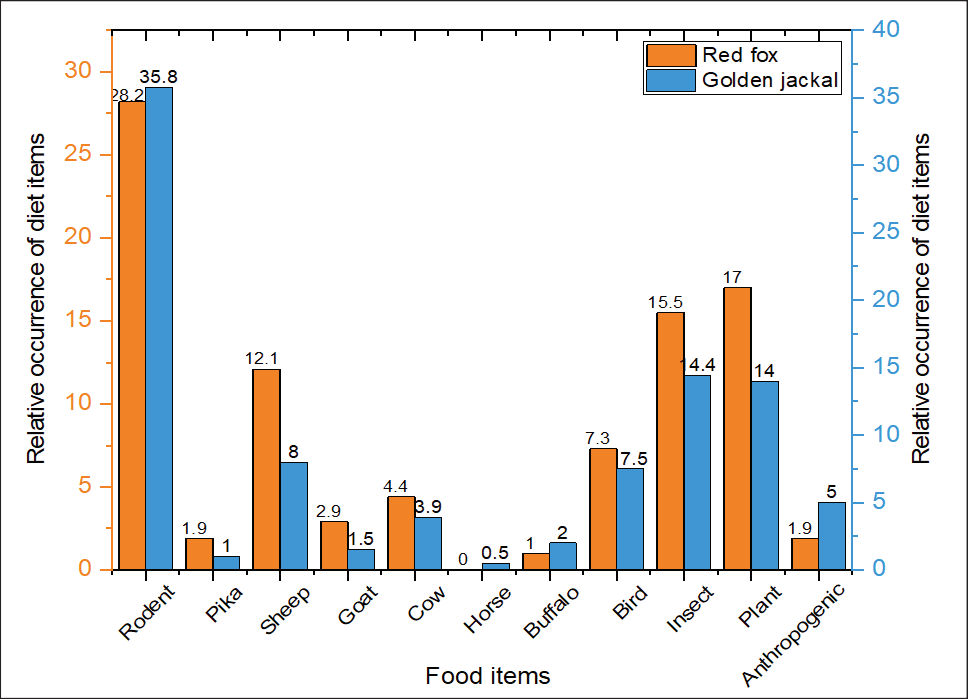
Relative occurrence of diet items in the scats of red fox and golden jackal in the Hirpora Wildlife Sanctuary.

**Figure 3.**
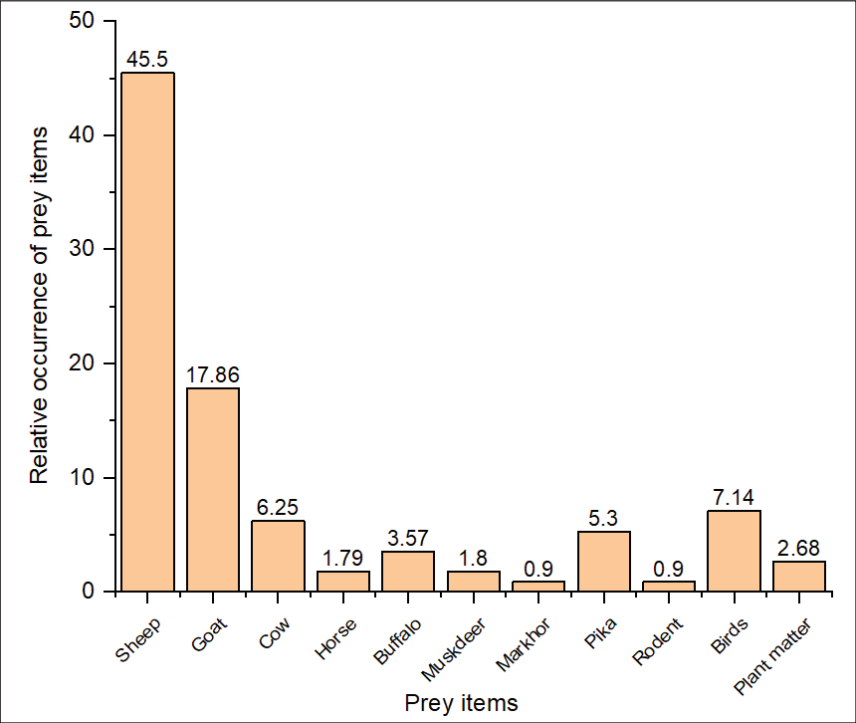
Relative occurrence of prey items in the diet of the Himalayan wolf in the Hirpora Wildlife Sanctuary.

We collected and examined 128 scat samples of golden jackals, 149 of red foxes, and 97 of Himalayan wolves. The analysis revealed the presence of 10 different types of dietary items in red fox scats, while golden jackal and Himalayan wolf scats contained 11 identified items each (Figs. 2, 3), along with unidentified material. The animal matter contributed more than the plant matter in all the canid species. The contribution of rodents was highest among all diet items with a relative occurrence (RO) of 28.2% and 35.8%, followed by livestock (sheep, goat, cow, horse, and buffalo) at 29.4% and 15.9% in the diet of the red fox and golden jackal, respectively (Fig. 2), whereas in the Himalayan wolf, domestic livestock contributed the most (RO: 74.97%) to the diet, followed by birds (RO: 7.14%) and wild ungulates (RO: 2.7%) (Fig. 3).

**Figure 4.**
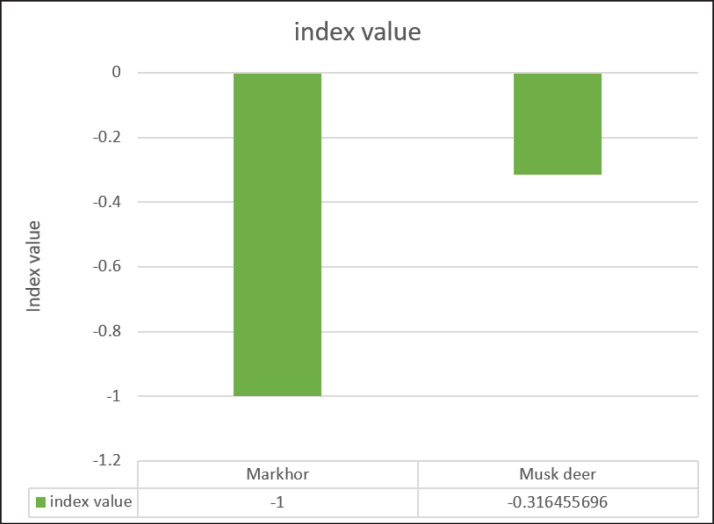
Jacob’s selectivity index value of two threatened ungulates by the Himalayan wolf in in the Hirpora Wildlife Sanctuary.

We did not find any evidence of wild ungulates in the scats of golden jackals and red foxes (Fig. 2); only Himalayan wolf scats showed traces of wild ungulates in the study area. The total count method revealed 18 individuals of the Pir Panjal markhor and 27 individuals of the Kashmir musk deer. Jacob’s selectivity index revealed that both the threatened ungulates were avoided by the Himalayan wolf. Markhor was avoided more than musk deer (Fig. 4).

**Figure 5.**
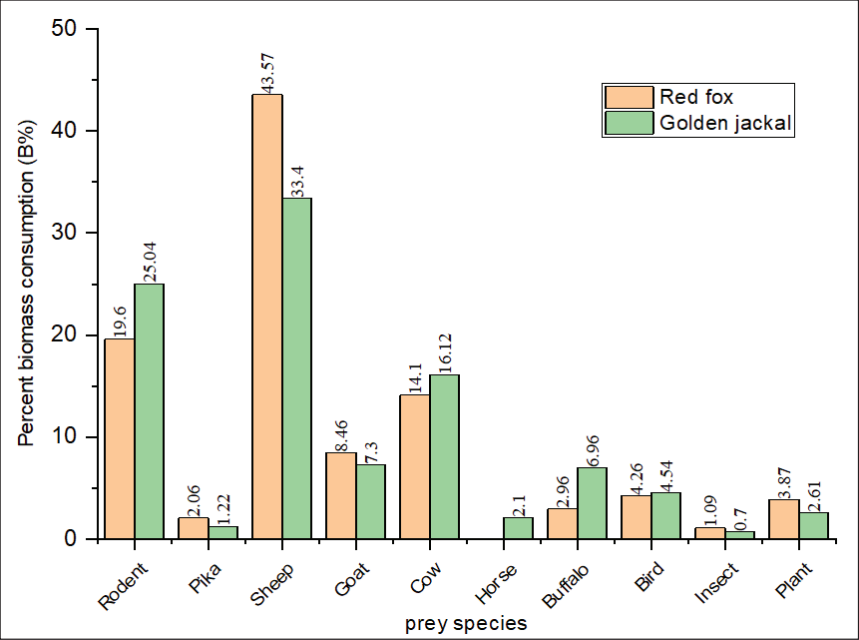
Biomass consumption of different prey items by red fox and golden jackal in the Hirpora Wildlife Sanctuary.

**Figure 6.**
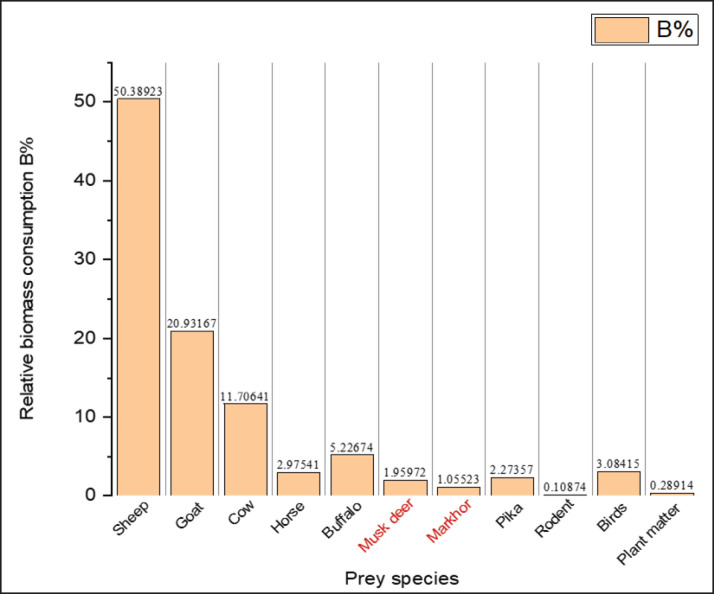
Biomass consumption of different prey items by the Himalayan wolf in the Hirpora Wildlife Sanctuary.

Domestic livestock contributed the highest, followed by rodents, in the biomass of red fox and golden jackal (Fig. 5). The percent biomass contribution of wild prey species was 8.77% in the Himalayan wolf. The rest, 91.25%, was contributed by domestic livestock. Among wild prey species, the two ungulates, musk deer and markhor, contributed collectively 3.01% (Fig. 6).

## Discussion

Our study provides valuable insights into the feeding habits and wild ungulate preferences of the wild canids in the sanctuary. Among the three wild canids, only the Himalayan wolf preys on wild ungulates. The traces recovered in scats confirmed the minimal role of wolves in the population dynamics of wild ungulates. Due to altitudinal separation and their affinity for human habitation, jackals used areas rarely frequented by wild ungulates in the sanctuary. Because of differences in body size, both ungulate species are unlikely prey for red foxes. However, red foxes can prey upon fawns or scavenge the carcasses of ungulates [34]. Foxes are better suited to hunting smaller prey that they can overpower more easily [35, 36]. For a red fox, the cost-benefit ratio likely does not favor pursuing large, difficult-to-catch animals when smaller, more manageable prey is available.

The ungulate estimation survey revealed that there is a small population of wild ungulates present in the sanctuary. Factors such as poaching, overgrazing by livestock, and development projects have all contributed to the decline of these ungulates in the area [21]. Human presence and disturbance in critical habitats can cause stress to wild ungulates. Frequent disturbances and the presence of livestock might lead to changes in behavior, lower reproduction rates, and an increased risk of contracting diseases [37, 38]. Changing climate patterns can indirectly affect ungulates by altering vegetation dynamics, food availability, and migration patterns [39].

The HWLS is under immense grazing pressure from the livestock of migratory herders [38, 40]. These large livestock populations expand and encroach on the critical habitats of wild ungulates. According to the current study, livestock serves as easy and plentiful prey for wild canids. The significant portion of livestock in the diet of wild canids can be attributed to the presence of migratory herders in the study area during the summer and autumn. Notably, heavy livestock depredation by wolves has been documented in the Trans-Himalayas of India [41]. Due to the scarce and scattered distribution of wild ungulates in the study area [21], their contribution to the wolves’ diet was nearly negligible. In regions where natural prey is limited, wolves supplement their diet by consuming domestic livestock [41, 42]. Conversely, in areas with abundant wild ungulate populations, wolves primarily feed on these natural prey species [37].

The Himalayan wolf is one of the potential predators of the wild ungulate species [18]. However, our results indicated that the two wild ungulates, Pir Panjal markhor and Kashmir musk deer, contributed very little to the diet of Himalayan wolves. This is probably due to the low availability of these ungulates resulting from their very low abundance and population in the study area. Such low numbers provide very little return for the high cost to a predator like the wolf. The thin population of wild ungulates in the region is a critical factor that may prompt the Himalayan wolf to adapt its foraging behavior and prey on larger-sized livestock to meet its dietary needs [37]. This scarcity likely compels the Himalayan wolf to descend to lower areas during the winter [42] in search of prey to fulfil its dietary requirements. The killing of livestock may create conflicts with the pastoral community, threatening the survival of the wolf.

Our results suggest that both ungulate species were avoided by the Himalayan wolf, but the wild musk deer was less avoided than the markhor. This could be due to various factors, such as differences in behavior, habitat use, or ease of predation. Markhor, which inhabit cliffs, might have developed adaptations that make them harder for predators like wolves to access. On the other hand, Kashmir musk deer, preferring alpine scrub areas, might be more vulnerable due to their habitat and behavior.

## Conclusion

Our research sheds light on the complex dynamics within the Himalayan ecosystem, particularly concerning the interactions between the wild canids (especially the Himalayan wolf), the wild ungulates, and the livestock in the HWLS. While the Himalayan wolf predation was initially considered a significant factor that might be affecting wild ungulate populations, our findings challenge this assumption and suggest that other factors, notably grazing pressure and human disturbance, may play substantial roles in shaping the ecological imbalance in the sanctuary, leading to the decline of wild ungulates. At the same time, the Himalayan wolves compensate for the diet by using easily available livestock, which could result in conflict with the pastoralists and locals. Our findings offer a valuable insight into the factors at play and provide a roadmap for future conservation strategies. By embracing ecosystem-level conservation approaches, implementing sustainable land management practices, engaging stakeholders, and applying relevant management actions based on science, we can work toward securing the future of both the iconic species within the sanctuary and the delicate balance of this remarkable ecosystem.

## List of Abbreviations

AMSL, Above Mean Sea Level; B%, Percent Biomass; COD, Coefficient of Digestibility; h, hour; HWLS, Hirpora Wildlife Sanctuary; IUCN, International Union for Conservation of Nature; m, meter; mm, millimeter. RO, Relative Occurrence.
